# Monitoring local inflammation in JIA

**DOI:** 10.1186/1546-0096-12-S1-I11

**Published:** 2014-09-17

**Authors:** Johannes Roth

**Affiliations:** 1 University of Münster, Münster, Germany

## 

Juvenile idiopathic arthritis (JIA) is a chronic inflammatory disease which commonly shows a remitting-relapsing course. Monitoring disease activity and adaptation of immune-suppressive therapy is still a challenge in clinical practice. In previous work our group has shown that members of the S100-protein family are reliable biomarkers for monitoring arthritis in JIA patients on medication and that these proteins may even predict risk of relapses in these patients. In my talk I will present novel data regarding the local release mechanism of these proteins during joint inflammation, use of serum concentrations of these S100-proteins for monitoring JIA as well as novel molecular imaging methods based on local S100-protein expression in preclinical models of inflammation and arthritis.

## Disclosure of interest

None declared.

